# Mind your step: Target walking task reveals gait disturbance in individuals with incomplete spinal cord injury

**DOI:** 10.1186/s12984-022-01013-7

**Published:** 2022-03-25

**Authors:** Freschta Mohammadzada, Carl Moritz Zipser, Chris A. Easthope, David M. Halliday, Bernard A. Conway, Armin Curt, Martin Schubert

**Affiliations:** 1 Spinal Cord Injury Center, Neurophysiology, Balgrist University Hospital, Forchstrasse 340, 8008 Zurich, Switzerland; 2 Department of Electronic Engineering, University of York, York, YO10 5DD UK; 3 York Biomedical Research Institute, University of York, York, UK; 4 Biomedical Engineering, University of Strathclyde, Glasgow, G4 0NW UK; 5 Cereneo Foundation, Center for Interdisciplinary Research, 6354 Vitznau, Switzerland

**Keywords:** Center of mass, Spinal cord injury, Gait, Visually guided walking, Balance control, Motor control, Locomotion

## Abstract

**Background:**

Walking over obstacles requires precise foot placement while maintaining balance control of the center of mass (CoM) and the flexibility to adapt the gait patterns. Most individuals with incomplete spinal cord injury (iSCI) are capable of overground walking on level ground; however, gait stability and adaptation may be compromised. CoM control was investigated during a challenging target walking (TW) task in individuals with iSCI compared to healthy controls. The hypothesis was that individuals with iSCI, when challenged with TW, show a lack of gait pattern adaptability which is reflected by an impaired adaptation of CoM movement compared to healthy controls.

**Methods:**

A single-center controlled diagnostic clinical trial with thirteen participants with iSCI (0.3–24 years post injury; one subacute and twelve chronic) and twelve healthy controls was conducted where foot and pelvis kinematics were acquired during two conditions: normal treadmill walking (NW) and visually guided target walking (TW) with handrail support, during which participants stepped onto projected virtual targets synchronized with the moving treadmill surface. Approximated CoM was calculated from pelvis markers and used to calculate CoM trajectory length and mean CoM Euclidean distance TW-NW (primary outcome). Nonparametric statistics, including spearman rank correlations, were performed to evaluate the relationship between clinical parameter, outdoor mobility score, performance, and CoM parameters (secondary outcome).

**Results:**

Healthy controls adapted to TW by decreasing anterior–posterior and vertical CoM trajectory length (p < 0.001), whereas participants with iSCI reduced CoM trajectory length only in the vertical direction (p = 0.002). Mean CoM Euclidean distance TW-NW correlated with participants’ neurological level of injury (R = 0.76, p = 0.002) and CoM trajectory length (during TW) correlated with outdoor mobility score (R = − 0.64, p = 0.026).

**Conclusions:**

This study demonstrated that reduction of CoM movement is a common strategy to cope with TW challenge in controls, but it is impaired in individuals with iSCI. In the iSCI group, the ability to cope with gait challenges worsened the more rostral the level of injury. Thus, the TW task could be used as a gait challenge paradigm in ambulatory iSCI individuals.

*Trial registration* Registry number/ ClinicalTrials.gov Identifier: NCT03343132, date of registration 2017/11/17.

**Supplementary Information:**

The online version contains supplementary material available at 10.1186/s12984-022-01013-7.

## Background

Incomplete spinal cord injury (iSCI) is associated with impaired ambulation [1]. Most individuals with iSCI can regain some walking ability, possibly allowing them to walk across unobstructed and varying surfaces without high risk of falling. However, they may experience difficulties when walking in more challenging environments, including outdoor terrains that are demanding in terms of limb coordination [2, 3], balance [4, 5], and attention [6], such as when negotiating obstacles [7] and adapting gait to changes in surface gradient [4].

However, the movement strategies applied to maintain adaptability in challenging walking conditions are poorly understood in iSCI and largely neglected in measures of rehabilitation progression.

Previous studies suggest that individuals with iSCI cannot fully adapt their gait pattern during challenging walking tasks [8]. An example of this occurs during inclined walking, where individuals with iSCI fail to maintain postural adaptations that adjust trunk and pelvis attitude to accommodate to the gradient change [4].

In contrast, while individuals with iSCI can successfully adapt gait for obstacle avoidance, they achieve this by adopting different kinematic strategies (increased knee flexion and greater trochanter height) to those observed in healthy controls [7]. Related to this, we have shown that individuals with iSCI retain capacity to modify temporal aspects of their gait pattern, including step time and double support phase, when body weight support is provided [9] or when changing walking speed [10]. Nevertheless, previous studies suggest that individuals with iSCI can have difficulties changing their gait pattern due to reduced hip and knee flexion, leading to decreased intralimb coordination [2], which may be related to their neurological level of injury (NLI) or spasticity [11].

We have conducted a comparative study on gait adaptation in individuals with iSCI and healthy controls performing a challenging walking task called Target walking (TW). Here, individuals adapt their gait pattern by stepping onto virtual targets projected onto a moving treadmill. TW requires visual guidance of foot placement to achieve modification of step length and width and is accomplished through anticipatory control strategies. To assess performance in this walking task, the objective measure of Center of Mass (CoM) movement is used.

A hypothetical focal CoM [12] represents the movement of the center of body mass during gait and can be a valuable tool for investigating comparative gait patterns in iSCI and control groups. Alterations in the three-dimensional CoM trajectories help to quantify changes due to pathological gait patterns, lack of stability, and adaptation strategies [13]. Depending on an individuals’ pathology, a specific strategy or compensation mechanism impacting CoM may allow that individual to avoid loss of balance or falls when walking over irregular terrain. While falls are more associated with reactive control (e.g., rapidly preventing falls by taking a step or grasping a handhold) [14], this study examines anticipatory (i.e., voluntary) control of foot placement making use of CoM measures to quantify the effectiveness of the adaptation (i.e., the ability to actively modulate ones gait pattern in response to, e.g., perturbations [15]) utilized by our participant groups. 

In clinical practice, skilled motor functions during daily life are assessed with the Spinal cord independence measure (SCIM III) outdoor mobility score (SCIM outdoor). SCIM III is an ordinal scale sensitive to changes in ambulation capability over time post injury and includes information on the dependence upon mobility aids [16]. In individuals with iSCI, walking abilities (e.g., walking distance) are largely determined by the severity of the injury and the NLI [17–19]. Depending on the NLI (tetraplegic or paraplegic), the injury will affect trunk stability [20, 21] and arm movement [22] to varying degrees which, together with the additive effects of impaired proprioception and voluntary motor control [23], will contribute to CoM movement alterations. For these reasons, we investigate CoM movement and its correlation with NLI in individuals with iSCI.

Our primary hypothesis was that individuals with iSCI, when challenged with TW, show a lack of gait pattern adaptability and this is reflected by impaired adaptation of CoM movement compared to healthy controls. We further hypothesized that gait adaptability, explored using the TW task, correlates with SCIM outdoor mobility score and NLI.

In healthy controls, changes in the excursions (e.g., reduced amplitude) of CoM trajectories from Normal walking (NW) to TW may indicate gait adaptability strategies used during TW. In individuals with iSCI, we report that CoM trajectory excursions are lost or attenuated, possibly caused by participants’ NLI, affecting dynamic trunk movement and intralimb coordination and limiting effective engagement of those compensation strategies used by healthy controls.

Our primary outcome was the difference in CoM trajectory length during TW compared to NW representing gait adaptability in individuals with iSCI compared to healthy controls.

The secondary outcome was the correlation between the clinical outcome measure, NLI, the outdoor mobility score and CoM trajectory length and mean Euclidean distance.

## Materials and methods

### Participants

Participants with iSCI were recruited from Balgrist University Hospital after signing informed consent. This trial was registered at ClincalTrials.gov (NCT03343132, date of registration 2017/11/17). The study clinicians assessed participants. All study clinicians were GCP (Good Clinical Practice)-trained and board-certified neurologists. Participants with iSCI and healthy controls were screened with standard history taking and clinical examination to exclude psychiatric or medical comorbidity. Inclusion criteria were subacute (3–6 months post-injury) or chronic (≥ 12 months post-injury) iSCI of any severity, etiology, and NLI, age ≥ 18 years, and ability to stand without physical assistance or handrails for > 120 s. The rationale to include subacute and chronic individuals with iSCI was to cover a broad range of walking abilities. Exclusion criteria were neurological diseases other than iSCI (e.g., multiple sclerosis, stroke), history of significant autonomic dysreflexia with treatment, and psychiatric or medical comorbidity interfering with following the task, orthopedic problems, heart insufficiency NYHA III-IV (New York Heart Association; heart insufficiency symptoms after light endurance, III, and permanent symptoms, IV), and dermatological issues (e.g., Decubitus) at the harness attachment site or clinicians concerns on treadmill (GRAIL) assessment (i.e., inability to complete the task). Neurological examination following International standards for neurological classification of spinal cord injury (ISNCSCI) [24] was performed on the day of enrollment in all participants determining sensorimotor incomplete ASIA impairment scale D accordingly. The sample size was determined based on previous studies at our institution that investigated gait in spinal cord injury [22], and individuals with iSCI were further included based on availability. Healthy controls were included if they were ≥ 18 years old, had no history of neurological disease or any other disease mentioned above, such as psychiatric or medical comorbidities, dermatological issues or orthopedic problems.

The single-center controlled diagnostic clinical trial was approved by the Zurich cantonal ethics Committee (BASEC-Nr. 2017-01780). All experiments were conducted in accordance with the Declaration of Helsinki.

### Experimental setup

Participants walked on a split-belt treadmill with two integrated force plates (GRAIL, Motek Medical B.V., Netherlands) at a self-selected walking speed with their own comfortable footwear. The self-selected speed was determined by increasing the treadmill speed slowly (0.1 m/s) while consistently getting feedback from the participants after each speed increment. To confirm that this speed reflects their preferred walking speed, treadmill speed was again increased by 0.1 m/s after and participants’ feedback requested. If the speed was too fast, it was decreased by 0.1 m/s. If the walking speed was too slow for the participants, the speed was increased by 0.1 m/s. This way, the participants could test a range of walking speeds and confirm what speed they were most comfortable with. Participants maintained each speed for at least 10 s before it was increased or decreased.

Participants were secured with a harness (JSP, Type PN21) that did not provide body weight support but would limit fall risk in the event of a participant losing balance. Similarly, participants were allowed to hold the handrails of the treadmill if needed. The participants completed the NW and TW tasks in random order (i.e., TW first or NW first).

Prior to data collection, participants were able to familiarize themselves with the NW task, TW task, and treadmill walking. Familiarization sessions were limited to one minute to avoid the risk of fatigue impacting on subsequent experimental sessions.

### Target walking

**Fig. 1:**
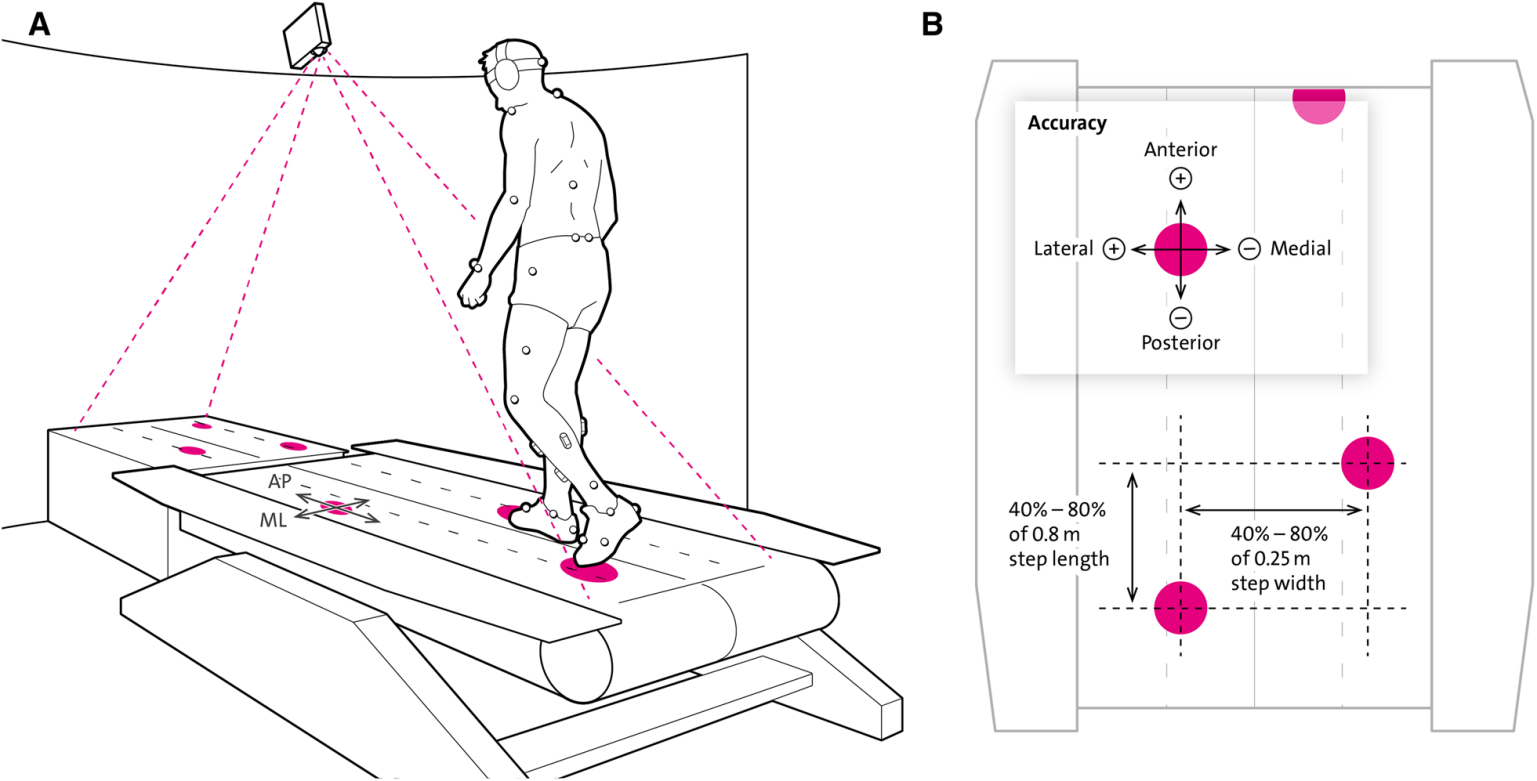
Target walking task. Schematic view of Motek GRAIL system. **A** Participants were asked to step onto moving white circular targets (10 cm diameter, here pink) projected on the black treadmill with their self-selected walking speed. **B** Aerial view of treadmill: participants had to change step length and width based on a variability of 40–80% of 0.8 m and 40–80% of 0.25 m, respectively, in order to step onto the targets. Accuracy was assessed in the anterior–posterior (AP) and medio-lateral (ML) direction monitored by a reflective marker placed on the second toe, the metatarsal 2 (MT2). Handrails and harness that were worn for safety are not shown in this schematic figure. This figure shows whole-body kinematics but for this study foot and pelvis markers were used

Participants were asked to step onto moving circular targets (white dots, diameter = 10 cm) projected onto and in front of the right and left treadmill belt (Fig. 1). The task duration was three minutes. This duration ensured that sufficient data and number of steps were available for our statistical analysis but short enough to ensure completion of the TW task in participants with iSCI and healthy controls.

The targets moved toward the participant at speed matched to the treadmill, giving the impression that the individual targets were “fixed” to the moving treadmill surface (Motek Forcelink, Amsterdam, the Netherlands, version 3.34.1). Targets were projected with a lead-in of greater than two stride lengths, allowing the participant to perceive and anticipate upcoming targets for at least the next three to four steps [25]. Stepping onto the targets required adjustments of step length (continuous uniform distribution randomization between 40 and 80% of 0.8 m step length) and step width (continuous uniform distribution randomization between 40 and 80% of 0.25 m step width).

Target reaching during foot placement was monitored by a 14 mm reflective marker placed on the base joint of the second toe—metatarsal 2 (MT2). Performance measures describing precise foot placement were calculated from the anterior–posterior (AP) and medio-lateral (ML) distance (error) between MT2 and the center of the target (Fig. 1B). To quantify ML errors, stepping medially from the center of the target was expressed by negative values, stepping laterally by positive values. Similarly, for AP errors stepping anteriorly from the center of the target resulted in positive values, and stepping posterior resulted in negative values.

Placement errors were calculated once per step at midstance. Calculated placement errors were not displayed for the participant but were recorded for offline analysis.

### Normal walking

In the NW task, no visual information was presented; participants were asked to look straight ahead and walk naturally for three minutes.

### Recordings

Foot and pelvis kinematics were assessed with a passive infra-red camera motion capture system (Vicon Motion Systems LTd, Oxford, UK) operating at 100 Hz and processed in Nexus 2.2.3 (Vicon, Oxford, UK) and custom-written MATLAB scripts (MATLAB R2017b). Markers at the heel and MT2 were used to assess the heel strike (HS) time and toe off (TO) time, respectively. Time of HS and TO were calculated by zero crossing in heel and toe marker velocity, respectively [26]. An approximated CoM was calculated by taking the midpoint between left posterior spina iliac to right anterior spina iliac and left anterior spina iliac to right posterior spina iliac [27, 28]. We did not use the full-body marker set and segment weights for CoM calculation because the harness prevented reliable placement of the torso markers, and the approximated CoM model is well understood in its limitations [27, 29, 30].

### Data analysis

CoM trajectories in anterior–posterior (AP), medio-lateral (ML), and vertical (V) directions were time-normalized to 100% gait cycle from HS to HS, using linear normalization with an anchor at the end of stance phase. The anchor was used to prevent warping of the trajectory through changes in relative stance and swing durations. Trajectories were then averaged for all recorded steps, centered by subtracting their mean and normalized by body height. For each walking condition, the following parameters were calculated from this trace. The total length of three 1-D trajectories for CoM, ${C}_{AP}$, ${C}_{ML}$, and ${C}_{V}$ were calculated in the AP-, ML- and V directions, respectively, with the summed Euclidean distance for each participant between sample points in time $i$ and number of sampling points $n$. ${C}_{AP}$ is defined by

1 $${C}_{AP}=\sum_{i=1}^{n-1}\sqrt{({{AP}_{i+1}-{AP}_{i})}^{2}}$$

1-D CoM trajectory lengths ${C}_{ML}$ and ${C}_{V}$ were calculated similarly.

The calculation of the total length of the 2-D projection of the CoM trajectory ${C}_{AP-ML}$ onto the AP-ML (transverse) plane was defined by

2 $${C}_{AP-ML}=\sum_{i=1}^{n-1}\sqrt{({{AP}_{i+1}-{AP}_{i})}^{2}+({{ML}_{i+1}-{ML}_{i})}^{2}}$$

Lengths of 2-D CoM projections onto the sagittal (${C}_{AP-V}$) and frontal (${C}_{ML-V}$) planes were calculated similarly. Likewise, the total length of the 3-D CoM trajectory length ${C}_{AP-ML-V}$ was calculated in the same way. The CoM trajectory length summarizes the extend of excursions and the total size of CoM movement.

To determine CoM shape and movement differences between TW and NW, mean Euclidean distances ${D}_{AP}$, ${D}_{ML}$, and ${D}_{V}$ in AP-, ML-, and V directions were calculated for each participant at each sample point $i$ in time and number of sampling points $n$. ${D}_{AP}$ is defined by

3 $${D}_{AP}=\frac{\sum_{i=1}^{n}\sqrt{({{AP}_{i;TW}-{AP}_{i;NW})}^{2}}}{n}$$

Mean 1-D Euclidean distances ${D}_{ML}$ and ${D}_{V}$ were calculated similarly.

The calculation of the mean 2-D Euclidean distance ${D}_{AP-ML}$ for AP-ML trajectory was defined by

4 $${D}_{AP-ML}=\frac{\sum_{i=1}^{n}\sqrt{({{AP}_{i;TW}-{AP}_{i;NW})}^{2}+({{ML}_{i;TW}-{ML}_{i;NW})}^{2}}}{n}$$

Mean 2-D and 3-D Euclidean distances ${D}_{AP-V}$, ${D}_{ML-V}$, and ${D}_{AP-ML-V}$ for trajectories AP-V, ML-V, and AP-ML-V were calculated similarly.

Since this is the first study using CoM during the TW task in participants with iSCI, we analyzed all CoM directions for a comprehensive characterization of their gait pattern. Mean number of analyzed gait cycles for NW in participants with iSCI was 152.3 (SD = 22.2) and in healthy controls 158.6 (SD = 14.2). For TW, the mean number of analyzed gait cycles was 163.3 (SD = 40.4) in participants with iSCI and in healthy controls 147.4 (SD = 35.6).

Hip, knee, and ankle angles for the cyclograms were time-normalized to 100% gait cycle such as the CoM trajectories, averaged, and then centered as described in Malik et al. [3].

Statistical analyses were performed using R version 3.6.1 (R Foundation for Statistical Computing, Austria) and Matlab (MATLAB R2017b). To compare time-, and height-normalized 1-D CoM trajectories between NW and TW, statistical parametric mapping (SPM) was used [31]. SPM calculations were performed in Matlab by implementing functions provided at www.spm1d.org (Version 2019). A nonparametric two-tailed paired t-test was used to separately compare the AP, ML, and V CoM trajectories during NW and TW. SPM(t) is the t-test statistic as a function of time and calculated separately at each time point. A critical threshold was computed that allowed inferences about significant differences in walking tasks (α = 0.05). Technical details and explanations are provided elsewhere [31, 32]. 

The Kolmogorov–Smirnov test was performed to assess data normality. The paired Wilcoxon-test (calculated by an exact method to calculate p-values in MATLAB based on the number of participants) was used to investigate CoM trajectory length differences for AP- and V directions between NW and TW in controls and participants as well as for the 2-D CoM AP-V trajectories and 3-D CoM trajectory AP-ML-V since most differences were observed there (p < 0.05). Furthermore, we used the non-paired Wilcoxon rank-sum test (using a MATLAB approximation method for calculating the p-value and reporting the z-statistic values due to sample size) to calculate the difference between controls and participants with iSCI in the mean Euclidean distance TW-NW of the AP- and V CoM trajectories, and the 2-D CoM AP-V trajectory as well as performance accuracy in the ML and AP direction between healthy controls and participants with iSCI. The Chi-square test of Independence was used to test for sex differences. Descriptive statistics were also used to compare walking speed, step length, and width within and between healthy controls and participants with iSCI. All reported p-values were Bonferroni-corrected. Results are shown as the median and interquartile range (IQR).

Spearman rank correlations were used to assess the relationship between 1. TW CoM trajectory lengths and task performance, 2. NW and TW CoM trajectory lengths and SCIM outdoor score, 3. NW and TW CoM trajectory lengths and NLI, 4. Mean Euclidean distance and SCIM outdoor score, and 5. Mean Euclidean distance and participants’ NLI. Spinal cord lesion height was numbered from 1 to 29, from C1 to S5. As a pragmatic approach, SCIM outdoor score was averaged from the acute to the chronic stage to account for the amount of recovery during rehabilitation. For instance, participants with a SCIM outdoor score of 8 in the acute phase of iSCI, which remained 8 in the chronic phase, might have developed a different gait pattern than participants with an initial lower score of 3 and recovery over time. In addition, following the time after the injury, other diseases, such as urinary tract infections, may have modified the gait pattern long-term and changed SCIM scores, respectively. Through averaging SCIM outdoor score, it was possible to consider current mobility (i.e., during the trial) while accounting for initial walking impairment and fluctuations (i.e., pain-related gait disturbances and iSCI-specific complications).

The primary outcome to determine gait adaptability was the difference in CoM trajectory length during TW compared to NW in individuals with iSCI and healthy controls.

The secondary outcome was the correlation between clinical outcome measure, outdoor mobility score and CoM trajectory length and mean Euclidean distance.

## Results

All measurements were completed without any adverse events (skin irritations/bruising) or safety incidents (falls/trips during treadmill walking). One participant with iSCI was excluded from the analysis because handrails were used in one condition but not the other, hence the tasks were not comparable. Another participant with iSCI was excluded from the analysis because of missing TW data.

Both right and left legs responded similarly; therefore, results are shown for the right leg only for lateralized parameters. In some cases, single parameters were not obtained, as reported in Table 1.

**Table 1:** iSCI demographics

ID	Age (y)	Height (cm)	Sex (m/f)	Walking speed (m/s)		Mean step length (m)		Mean step width (m)		ML accuracy (cm)	AP accuracy (cm)	AIS	LEVEL of injury	Time since injury (y)	Type of Injury	LEMS score		Mean SCIM (max 8)	Walking aids
				NW	TW	NW	TW	NW	TW							R	L		
01	79	170	m	0.35	0.3	0.24	0.35	0.17	0.16	− 1.53	4.0	D	L3	24	Traumatic	20	22	na	Canes
02	42	173	m	0.4	0.4	0.33	0.34	0.13	0.16	na	na	D	C3	1	Degenerative	25	25	5	No
03	65	175	m	1.2	1	0.58	0.45	0.09	0.18	− 0.23	7.72	D	C4	1	Traumatic	25	25	6	No
04	57	176	m	0.8	0.8	0.52	0.39	0.09	0.16	− 1.75	6.68	D	T4	0.3	Traumatic	25	25	7	No
06	36	180	m	0.8	0.8	0.53	0.43	0.17	0.19	− 0.27	6.10	D	T7	5	Traumatic	25	25	5	No
07	69	167	f	0.6	0.6	0.36	0.40	0.19	0.2	− 0.45	6.11	D	C5	6	Traumatic	23	23	3	No
09	48	182	m	0.9	0.8	0.58	0.40	0.15	0.17	− 3.0	11.50	D	L3	18	Traumatic	24	24	7	No
10	58	163	f	0.8	0.6	0.54	0.41	0.14	0.18	− 0.73	7.94	D	T4	2	Toxic	25	25	1	Wheelchair
11	58	170	f	1	1	0.61	0.40	0.11	0.16	− 1.30	9.01	D	T7	11	Degenerative	25	25	8	No
13	70	170	m	0.8	0.7	0.52	0.41	0.15	0.20	− 2.38	7.59	D	T4	7	Tumor	25	25	4	No
14	66	170	m	0.8	0.7	0.44	0.39	0.15	0.16	− 0.93	5.54	D	T3	14	Tumor	25	25	8	No
15	67	177	m	0.55	0.6	0.55	0.43	0.09	0.17	− 1.73	1.03	D	L1	8	Tumor	19	16	6	Canes
16	54	184	m	0.7	0.6	0.55	0.40	0.14	0.16	− 0.76	5.44	D	T12	6	Traumatic	25	25	5	No
Median	58	173		0.8	0.7	0.53	0.4	0.14	0.17	− 1.12	6.4			6		25	25	5.5	
IQR	13	7.8		0.2	0.2	0.14	0.03	0.05	0.02	1.15	2.34			10		1.25	1.25	2.5	
Stats				p = 0.02		p = 0.006		p < 0.001											

*NW* normal walking, *TW* target walking, *m* male, *f* female, *ML* medio-lateral, *AP* anterior–posterior, *AIS* American Spinal Injury Association (ASIA) impairment scale, *LEMS* lower extremity motor score, *SCIM* spinal cord independence measure, *R* right, *L* left, *IQR* interquartile range, *y* years, *Stats* statistics, *na* not available; paired Wilcoxon test

### Participants

Thirteen participants with subacute and chronic iSCI (ten males; median = 58 years, IQR = 13, one subacute and twelve chronic participants with iSCI) were enrolled in this study. Twelve healthy controls (six males; median = 27 years, IQR = 6) without a history of neurological disease were enrolled in this study. Participants with iSCI were older than healthy controls (p < 0.001). All participants with iSCI had preserved or reacquired walking ability, most participants regularly walked without walking aids. Three participants used walking aids outdoors but could stand independently without support in everyday life and consequently made use of the handrails during the NW and TW task. Healthy controls did not hold the handrails. Tables 1 and 2 list all details on participants’ demographics and characteristics.

### Gait parameters

The median self-selected comfortable walking speed across all healthy controls in NW was 1 m/s (IQR = 0.2) which was significantly different (T = 78, p < 0.001) from their median TW speed of 0.9 m/s (IQR = 0.15) (Table 2). In participants with iSCI, the median preferred walking speed during NW of 0.8 m/s (IQR = 0.2) was not significantly different (T = 34.5, p = 0.02; p < 0.012, p-adjust) from the median walking speed of 0.7 m/s (IQR = 0.2) during TW (Table 1). Between groups, walking speed was significantly different in both NW (T = 214, z = 3.16, p = 0.002) and TW (T = 206.5, z = 2.75, p = 0.006). Furthermore, in both groups, we found significant differences between NW and TW in median step length (iSCI, T = 82.5, p = 0.006; controls, T = 78, p < 0.001) and in step width (iSCI: T = 2, p < 0.001; controls: T = 0, p < 0.001). These differences were expected as both step length and width were externally imposed by the TW task condition (Fig. 1). Between participants with iSCI and controls, step width during TW was different (T = 217.5, z = 3.36, p < 0.001) but not during NW (T = 146, z = − 0.52, p = 0.6). Step length during NW (T = 204.5, z = 2.62, p = 0.014; p < 0.012, p-adjust) and TW (T = 160, z = 0.21, p = 0.8) did not differ between participants with iSCI and controls. The Chi-square test shows that sex and type of participants group (iSCI/healthy control) are independent (${\chi }^{2}(1)=$ 1.96, p = 0.161).

**Table 2:** Participant demographics

ID	Age (years)	Height (cm)	Gender (m/f)	Walking speed (m/s)		Mean step length (m)		Mean step width (m)		ML Accuracy (cm)	AP Accuracy (cm)
				NW	TW	NW	TW	NW	TW		
01	25	160	f	1.2	1.1	0.64	0.40	0.10	0.20	0.01	8.41
02	21	166	f	0.95	0.7	0.61	0.40	0.13	0.25	− 0.51	6.68
03	32	168	m	0.95	0.9	0.58	0.41	0.13	0.23	− 0.36	6.55
04	26	176	m	1.1	1	0.67	0.40	0.10	0.17	− 0.60	6.99
05	31	150	f	0.8	0.7	0.51	0.42	0.13	0.21	0.23	5.14
06	29	174	m	1	0.9	0.54	0.40	0.13	0.19	0.22	8.45
07	27	173	m	1	0.9	0.58	0.40	0.10	0.19	− 0.76	5.70
08	34	178	m	1.3	1.1	0.71	0.40	0.14	0.2	0.14	5.63
09	26	160	m	0.85	0.8	0.53	0.41	0.23	0.26	0.08	5.75
10	27	170	f	1.25	1	0.65	0.41	0.14	0.19	0.07	9.23
11	32	162	f	1	0.9	0.55	0.40	0.16	0.24	0.57	5.98
12	23	170	f	1	0.9	0.62	0.40	0.14	0.25	0.34	5.58
Median	27	169		1.0	0.9	0.59	0.40	0.13	0.20	0.08	6.27
IQR	6	13		0.2	0.15	0.10	0.01	0.03	0.06	0.66	2.04
Stats				p < 0.001		p < 0.001		p < 0.001			

*NW* normal walking, *TW* target walking, *m* male, *f* female, *AP* anterior–posterior, *ML* medio-lateral, *IQR* interquartile range, *stats* statistics; paired Wilcoxon test

### Center of mass trajectories

**Fig. 2:**
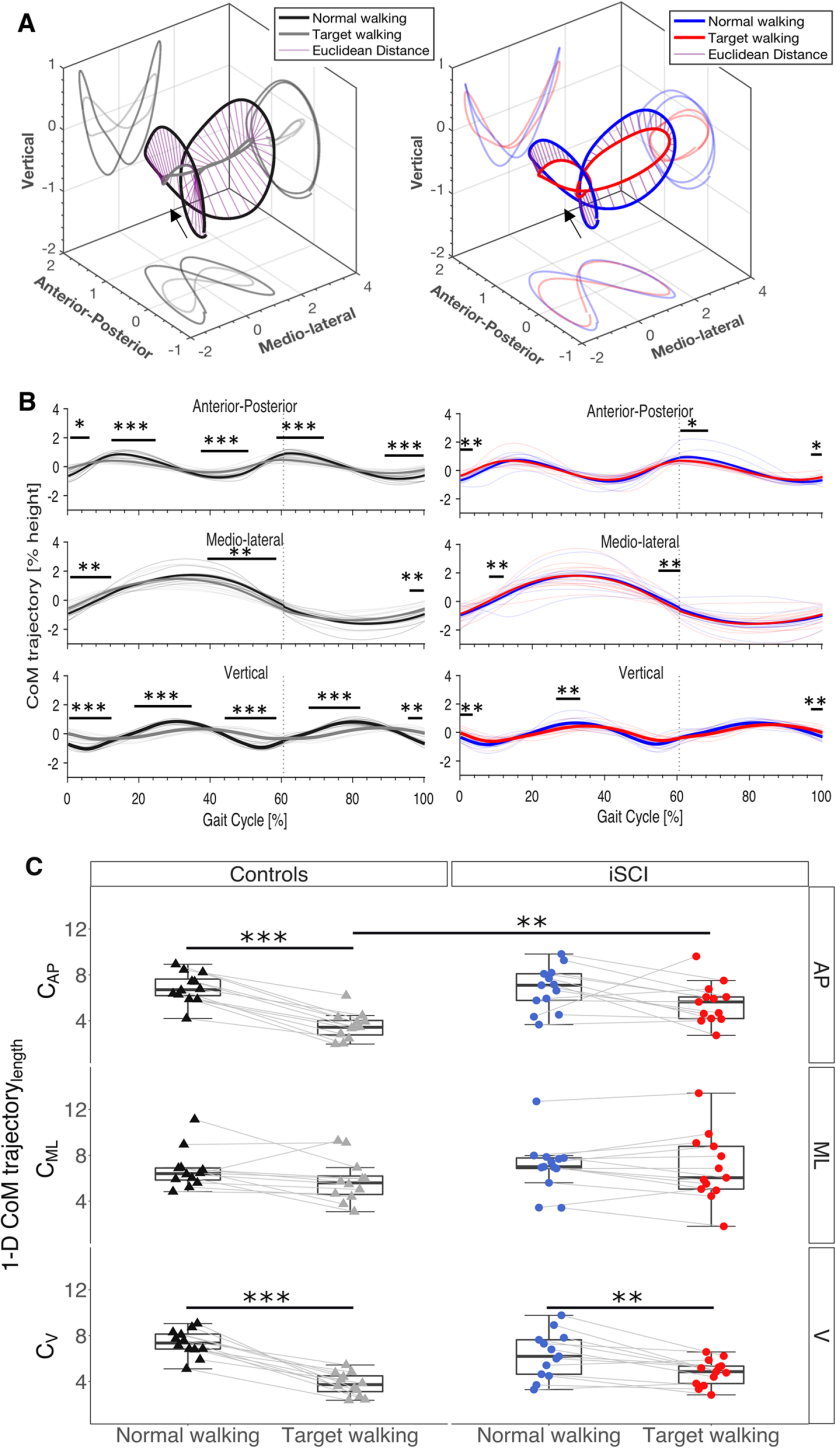
Center of Mass trajectories. **A** CoM 3-D and 2-D trajectories in TW are reduced in participants with iSCI (top, right) and controls (top, left); however, the reduction is more pronounced in controls (top, left). Arrows indicate movement direction. **B** Reductions may be caused primarily by TW CoM trajectories in AP and V directions (middle) in participants with iSCI (middle, right) and controls (middle, left). Differences between NW and TW are significant at, and following HS and TO for participants and for controls throughout almost the entire CoM trajectory. TW induced alterations of CoM trajectory are assumed to subserve an increase of inertial stability of CoM and further to optimize CoM movement for accurate foot placement. For redundancy reasons, only differences in CoM trajectory lengths are shown and omitted for CoM 3-D and 2-D trajectories. Gray dashed lines depict time of TO. **C** CoM trajectory length is reduced most consistently in the V direction in controls and participants with iSCI. Participants with iSCI show on average less reduction of CoM trajectory length and more variability in AP, ML, and V when performing TW. Dots and triangles represent single participants. *p < 0.05; **p < 0.01; ***p < 0.001. *CoM* center of mass, *TW* target walking, *NW* normal walking, *AP* anterior–posterior, *ML* medio-lateral, *V* vertical, *iSCI* incomplete spinal cord injury, *HS* heel strike, *TO* toe off

Averaged, centered and height-normalized 3-D CoM movements were greater during NW than TW in controls and participants with iSCI (Fig. 2A). However, controls seem to have adapted more successfully to the TW condition than participants with iSCI by reducing CoM movement. The SPM t-test indicated significant differences of AP-, ML-, and V CoM trajectories between TW and NW in controls and participants with iSCI (Fig. 2B). The strongest TW effects, which were exacerbated in controls compared to participants with iSCI, were observed primarily in the AP- and V CoM trajectories (Fig. 2B, Table 3). These can partially be attributed to a vertical shift of TW CoM trajectory from NW CoM trajectory.

**Table 3:** CoM trajectory differences between NW and TW throughout the gait cycle

	Stance phase						Swing phase					
	AP		ML		V		AP		ML		V	
	%	p-value	%	p-value	%	p-value	%	p-value	%	p-value	%	p-value
iSCI	0–6	< 0.01	8–12	< 0.01	0–6	< 0.01	60–68	< 0.05			98–100	< 0.01
			55–64	< 0.01	26–34	< 0.01	98–100	< 0.05				
Controls	0–4	< 0.05	0–12	< 0.01	0–10.5	< 0.001	56–76	< 0.001	90–100	< 0.01	64–84	< 0.001
	10–26	< 0.001	40–60	< 0.01	18–39	< 0.001	84–100	< 0.001			96–100	< 0.01
	36–52	< 0.001			41–59	< 0.001						

*NW* normal walking, *TW* target walking, *CoM* center of mass, *AP* anterior–posterior, *ML* medio-lateral, *V* vertical, *iSCI* incomplete spinal cord injury, *%* percentage of gait cycle

CoM length of AP- and V trajectories during NW were significantly increased compared to TW CoM length in healthy controls (${C}_{AP}$: T = 78, p < 0.001 and ${C}_{V}$: T = 78, p < 0.001; p < 0.0042, p-adjust). In participants with iSCI, only the V CoM trajectory was increased during NW compared to TW (${C}_{V}$: T = 86, p = 0.002; p < 0.0042, p-adjust) (Fig. 2C). During TW, significantly greater CoM AP trajectory length was found for participants with iSCI than controls (${C}_{AP}:$ T = 102, z = − 2.91, = 0.004; p < 0.0042, p-adjust). For redundancy reasons, statistical tests were not done for 2-D and 3-D CoM trajectories since the results will most likely be driven by the differences found in the axial CoM trajectories shown in Fig. 2B and C. Mean Euclidean distance was only different in the V CoM trajectory between participants with iSCI and controls (${D}_{V}$: T = 210, z = 2.91, p = 0.004; p < 0.02, p-adjust).

### Relationship between CoM trajectory, SCIM outdoor mobility score, and NLI

**Fig. 3:**
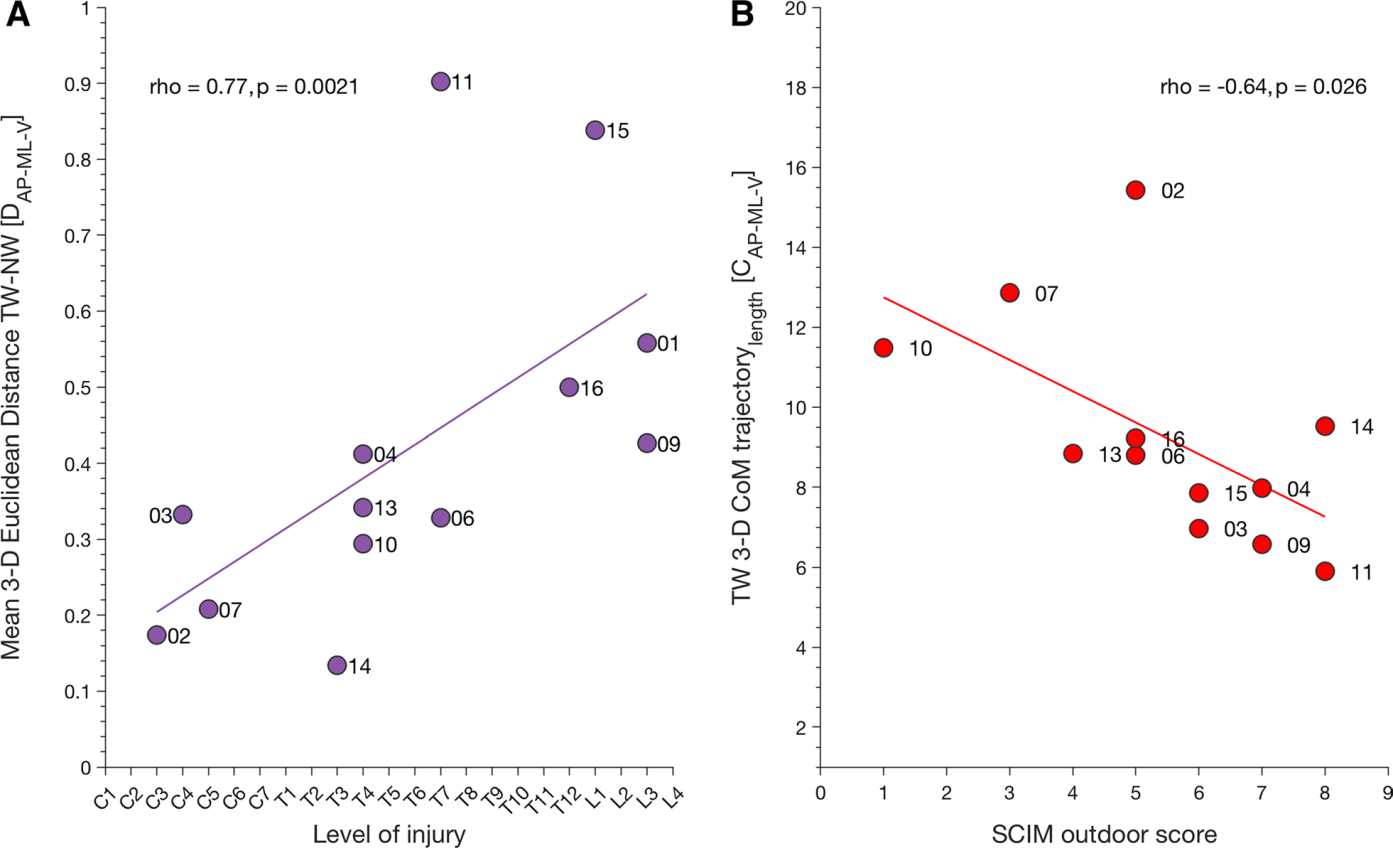
Relationship between CoM and clinical parameter and outdoor mobility score. **A** Ability of participants with iSCI to reduce 3-D CoM movement during TW is related to their lesion level (as well as 1-D AP trajectory and 2-D trajectories, see “Results”), as depicted by the mean Euclidean distance between TW and NW. **B** Mean SCIM outdoor mobility score is inversely correlated to the length of 3-D CoM trajectory length (also AP 1-D trajectory, and ML-AP 2-D trajectory, see “Results”) when participants with iSCI perform TW suggesting a relation with their lack of adaptability to adjust to the task. Dots represent single participants and their ID. *CoM* center of mass, *TW* target walking, *NW* normal walking, *AP* anterior–posterior, *ML* medio-lateral, *V* vertical, *SCIM outdoor* spinal cord independence measure outdoor mobility, *iSCI* incomplete spinal cord injury

We obtained a positive correlation between level of injury and mean 3-D Euclidean distance between TW and NW (${D}_{AP-ML-V}:$ spearman’s rho(11) = 0.76, p = 0.002) (Fig. 3A). The higher the mean 3-D Euclidean distance, the better the participants with iSCI reduced their CoM movement during TW compared to NW. Participants with an injury at cervical segments seemed to be less able to adapt their gait patterns than participants with thoracic or lumbar NLI. Thus, coping with the demands implied by the TW task seems to be dependent on NLI. Similar positive correlations were found between NLI and mean Euclidean distance in AP trajectory (${D}_{AP}:$ spearman’s rho(11) = 0.84, p < 0.001), AP-ML 2-D trajectory (${D}_{AP-ML}:$ spearman’s rho(11) = 0.8, p < 0.001), AP-V 2-D CoM trajectory (${D}_{AP-V }:$ spearman’s rho(11) = 0.77, p = 0.002). NLI did not correlate with TW 3-D CoM trajectory length (${C}_{AP-ML-V}:$ spearman’s rho(11) = − 0.26, p = 0.39), NW 3-D CoM trajectory length (${C}_{AP-ML-V}:$ spearman’s rho(11) = 0.08, p = 0.79), nor with 2-D CoM trajectory lengths during TW (${C}_{AP-ML}:$ spearman’s rho(11) = − 0.32, p = 0.28; ${C}_{AP-V}:$ spearman’s rho(11) = 0.22, p = 0.47; ${C}_{ML-V}:$ spearman’s rho(11) = − 0.52, p = 0.07) and NW (${C}_{AP-ML}:$ spearman’s rho(11) = − 0.01, p = 0.99; ${C}_{AP-V}:$ spearman’s rho(11) = 0.43, p = 0.14; ${C}_{ML-V}:$ spearman’s rho(11) = − 0.16, p = 0.61). Similarly, NLI did not correlate with 1-D CoM trajectory lengths during TW ${(C}_{AP}:$ spearman’s rho(11) = 0.35, p = 0.27; ${C}_{ML}:$ spearman’s rho(11) = − 0.5, p = 0.09; ${C}_{V}:$ spearman’s rho(11) = − 0.12, p = 0.73) or NW (${C}_{AP}:$ spearman’s rho(11) = 0.52, p = 0.07; ${C}_{ML}:$ spearman’s rho(11) = − 0.22, p = 0.47; ${C}_{V}:$ spearman’s rho(11) = 0.1, p = 0.75).

SCIM outdoor mobility score is a valuable measure of functional ability outside where complex ground conditions require that gait has to be adapted to the presence of obstacles and prevailing surround conditions. As seen in Fig. 3B, the 3-D CoM length of AP-ML-V trajectory obtained in TW negatively correlated with the mean SCIM outdoor mobility score (${C}_{AP-ML-V}:$ spearman’s rho(11) = − 0.64, p = 0.026). Thus, participants with low mean SCIM outdoor mobility scores exhibited increased CoM movement in the challenged gait condition. Similar results were found for AP trajectory (${C}_{AP}:$ spearman’s rho(11) = − 0.65, p = 0.02) and AP-ML trajectory (${C}_{AP-ML}:$ spearman’s rho(11) = − 0.64, p = 0.03). Mean SCIM outdoor mobility score did not correlate with TW 3-D Euclidean distance (${D}_{AP-ML-V}:$ spearman’s rho(11) = 0.32, p = 0.31), 2-D Euclidean distances (${D}_{AP-ML}:$ spearman’s rho(11) = 0.19, p = 0.53; ${D}_{AP-V}:$ spearman’s rho(11) = 0.33, p = 0.29; ${D}_{ML-V}:$ spearman’s rho(11) = 0.11, p = 0.73) nor 1-D Euclidean distances (${D}_{AP}:$ spearman’s rho(11) = 0.19, p = 0.55; ${D}_{ML}:$ spearman’s rho(11) = − 0.15, p = 0.64; ${D}_{V}:$ spearman’s rho(11) = 0.51, p = 0.09).

### TW task performance

To examine whether CoM trajectory lengths were related to task performance, we investigated the relationship between placement accuracy in target stepping and TW CoM trajectory length.

Participants (median = − 1.1 cm, IQR = 1.1) and controls (median = 0.08 cm, IQR = 0.7) stepped close to the center of the target in ML direction. However, participants with iSCI generally stepped more medially to targets than the controls as indicated by the negative performance values (Table 1; Additional file 1: Fig. S1A); ML accuracy was significantly different between controls and participants with iSCI (T = 209.5, z = 3.41, p < 0.001). In participants with iSCI and healthy controls, no relationship was found between ML accuracy and TW 1-D CoM length of V trajectory (iSCI, ${C}_{V}$: spearman’s rho(11) = 0.46, p = 0.13; controls, ${C}_{V}$: spearman’s rho(22) = − 0.4, p = 0.2) (Additional file 1: Fig. S1A). In the AP direction, overestimation of the center of the target by both participants with iSCI (median = 6.4 cm, IQR = 2.4) and controls (median = 6.3 cm, IQR = 2.1) was found (Tables 1 and 2; Additional file 1: Fig. S1B). AP Accuracy and TW 2-D CoM AP-V trajectory length showed a negative correlation for participants with iSCI (${C}_{AP-V}$: spearman’s rho(11) = − 0.8, p = 0.003); no significant correlation was found for healthy controls (${C}_{AP-V}$: spearman’s rho(22) = − 0.48, p = 0.12) (Additional file 1: Fig. S1B).

### Effects of walking speed on CoM

**Fig. 4:**
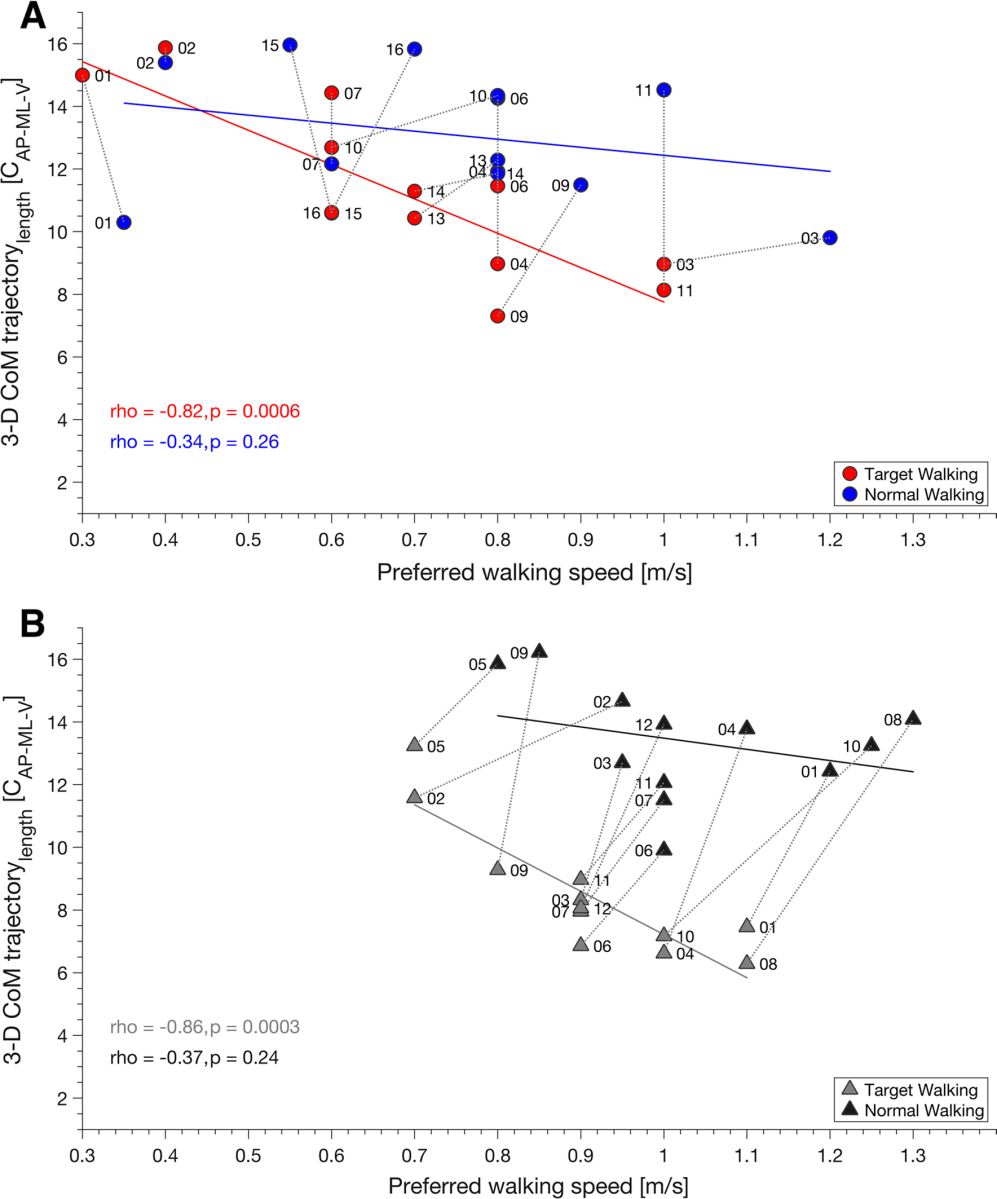
CoM trajectory length is related to preferred walking speed in controls and participants with iSCI. The relationship between preferred walking speed and CoM 3-D trajectory length is changed during TW (red dots) in participants with iSCI (**A**) and controls (gray triangles) (**B**) while the latter shows more decline and greater reduction than the former. In participants with iSCI, more variation of walking speed is obtained. Some participants, e.g., P01, P02, P07, and P15 prefer lower walking speeds during both NW and TW (A). The clear separation of TW and NW CoM 3-D trajectory length found in both participants and controls at similar walking speeds may be seen as an indication that reduction of CoM 3-D trajectory length is a common strategy to cope with TW independent of pathology and independent of preferred walking speed. Gray colored dotted lines connect NW and TW for each participant. Dots and triangles represent single participants and their ID. *AP* anterior–posterior, *ML* medio-lateral, *V* vertical, *TW* target walking, *NW* normal walking

While walking speed in TW was negatively correlated with 3-D CoM trajectory length in participants with iSCI (spearman’s rho(11) = − 0.82, p < 0.001) (Fig. 4A) and healthy controls (spearman’s rho(22) = − 0.86, p < 0.001) (Fig. 4B), this relation was not observed during NW. Thus, 3-D CoM trajectory length reduction during TW could not be explained by a variation of walking speed alone because TW 3-D CoM trajectory length decreased disproportionately with increasing walking speed compared to similar walking speed during NW. Walking speed in participants with iSCI was generally lower, and accompanied by an increase in inter-subject variability. Four participants (P01, P02, P07, P15) preferred lower walking speeds during NW and TW (Fig. 4A) in contrast to controls (Fig. 4B).

Significant correlations between TW walking speed and TW CoM trajectory lengths were found for participants with iSCI (${C}_{AP}$: spearman’s rho(11) = − 0.79, p = 0.001; ${C}_{ML}$: spearman’s rho(11) = − 0.67, p = 0.01; ${C}_{AP-V}$: spearman’s rho(11) = − 0.68, p = 0.01;${C}_{AP-ML}$: spearman’s rho(11) = − 0.85, p < 0.001; ${C}_{ML-V}$: spearman’s rho(11) = − 0.61, p = 0.03) and controls (${C}_{AP}$: spearman’s rho(22) = − 0.67, p = 0.02; ${C}_{ML}$: spearman’s rho(22) = − 0.88, p < 0.001; ${C}_{AP-ML}$: spearman’s rho(22) = − 0.88, p < 0.001; ${C}_{ML-V}$: spearman’s rho(22) = − 0.78, p = 0.002). NW walking speed correlated with NW 1-D CoM trajectory length in ML direction in participants with iSCI (${C}_{ML}$: spearman’s rho(11) = − 0.6, p = 0.03) and controls (${C}_{ML}$: spearman’s rho(22) = − 0.6, p = 0.04). Results and Figure of qualitative analyses of cyclograms can be found in the Additional file 1: Fig. S2.

## Discussion

### Summary of main findings

The main hypothesis was that individuals with iSCI lack the ability to adapt their gait pattern during a gait challenge requiring visually guided foot placement (TW) which is reflected by an impaired adaptation of CoM movement control compared to healthy controls. Thus, this study analyzed CoM movement control during TW and compared this to a regular treadmill walking task (NW) in participants with iSCI and healthy controls. Adaptability strategies during TW were similar in participants with iSCI and healthy controls, involving a disproportionate reduction of CoM trajectory length, which exceeded reductions systematically observed during NW. However, participants with iSCI were less able to adjust to the TW task, and the extent of this reduced capacity corresponded with the outdoor mobility score: 3-D CoM trajectory length obtained during TW correlated with mean SCIM outdoor score. Furthermore, the mean Euclidean distance in the 3-D CoM trajectory was related to the participants’ NLI, indicating that gait adaptability was worse the more rostral the NLI. We will discuss these results in the clinical context of iSCI and with respect to its functional significance.

### Coping strategy observed in TW

In this study, TW introduced a voluntary modification of step length and width by visually guiding foot positioning. This condition is characteristic of foot placement in outdoor environments. All participants with iSCI were able to complete the task. However, healthy controls strongly reduced their CoM trajectories during TW task in AP- and V directions (Fig. 2C), whereas participants with iSCI only adapted in the V direction. Voluntary control is actively needed to stabilize dynamic movements to accomplish the TW task actively; thus, there may be a tradeoff between safety/stability and target stepping accuracy. While AP accuracy was similar in participants with iSCI and healthy controls, differences were only observed for ML accuracy. Though no significant relationship was found between V CoM trajectory length and ML accuracy, we assume that the reduction of V CoM may be a strategy to maintain stability in the TW task. Seemingly high ML- and AP accuracy in participants with iSCI was contradicted by altered CoM trajectory length and mean Euclidean distance, indicating insufficient adaptation mechanisms. The TW task was selected among other established walking tasks, such as stepping over obstacles [7] or inclined walking [4], because it entails adaptive and anticipatory strategy selection and can be integrated into treadmill based gait training protocols. The CoM movement is meaningful, since it measures body movement in different directions, and objectively assesses balance.

Walking speed is an acknowledged clinical outcome measure that quantifies walking capacity and improvement over time in individuals with iSCI [33–35]. It is also known to influence CoM, especially in the ML trajectory [36], as it decreases with increasing speed [37], a relationship we found in participants with iSCI and healthy controls during TW and NW (for NW only in ML trajectory). Furthermore, walking at higher speeds may be associated with a more stable forward gait since the CoM stays within the base of support and prevents one from falling [38]. Conversely, participants with iSCI in this and previous cohorts tend to walk slower [39, 40], decrease step length [41], regulate walking demands, and maintain gait stability [42]. In this study, we have shown that, indeed, most participants and all healthy controls reduce their preferred walking speed to adjust to the TW demand (Fig. 4). However, despite the similar range of walking speeds during NW and TW, CoM trajectory lengths were different. Thus, reduction of CoM trajectory length during TW may be related to a common strategy employed to cope with TW demands, independent of pathology and preferred walking speed. However, functional restrictions reflected in the NLI and outdoor mobility score found in participants with iSCI might dampen the usage of this common strategy.

### Interrelation of NLI, outdoor mobility score, and gait disturbances

Participants with iSCI and high SCIM outdoor mobility scores could reduce TW 3-D CoM trajectory length better than those with a lower score (Fig. 3B). Therefore, CoM trajectory length in TW may reflect community walking abilities. Furthermore, CoM might be more responsive to subtle changes over time than SCIM outdoor score, because the maximum SCIM score of 8 indicating “Walking without aids” entails a ceiling effect.

A possible explanation might be the reduced possibility for dynamic involvement of trunk muscles, counter-motion arm swing, and inter-segmental coordination, which prevail in high NLIs due to the craniocaudal extension of spinal cord network disturbance and that could not be reflected by the CoM trajectory length. This will reduce the capacity for dynamic sensory-motor adaptation during gait [4, 22, 43] and limit the integration of proprioceptive and exteroceptive input to compensate for challenges when walking [44] in individuals with iSCI. In addition, those participants with lumbar lesions might have more peripheral involvement in their injury compared to cervicothoracic levels. Therefore less spasticity may alter gait patterns [11], notwithstanding that disruption of spinal networks at more cranial levels seriously impedes walking abilities.

### Limitations

This study included a heterogeneous group of 13 participants with iSCI with respect to injury mechanisms, NLIs, and central and peripheral neural pathologies, which each per se will influence gait pattern. There was no systematic bias from the different pathologies because CoM trajectories were not affected by traumatic (7/13) or non-traumatic injury (6/13) presentation. Controls were not age-matched, and we acknowledge that age could affect the differences found in controls and participants with iSCI since it influences gait stability [45, 46] and ML stability decreases with age [47]. However, the neurological impairment of participants with iSCI markedly exceeded changes that could be attributed to age.

Similarly, walking speed was not matched between controls and participants with iSCI. Walking speeds were chosen by participants individually, which allows the most personalized gait investigation possible. The walking tasks were not adapted to individual step length and width. While this may have worsened performance, particularly in the AP direction because of the moving treadmill, it can be assumed to introduce challenges comparable to natural environments and affect participants with iSCI and controls similarly. Furthermore, three participants with iSCI held the handrails during the tasks, which influences CoM by providing balance support [48]. In this cohort, however, we did not find an effect of handrail use on walking since there was no difference in the CoM movement compared to the other participants. Furthermore, we did not assess balance scales such as the activities-based balance scale [49]. To validate the utility of the TW task to assess motor strategy selection following iSCI, a comprehensive assessment across a larger group of iSCI individuals with varying clinical characteristics, with regard to spasticity, muscle weakness, and limb coordination and age-matched control group is still needed. Lastly, as participants only engaged in single experimental sessions, statements about the reliability and sensitivity of the TW task cannot be made. Future studies should consider testing the inter-session reproducibility of TW tasks.

## Conclusions

This study used a TW task to investigate gait differences between individuals with iSCI and healthy controls. Individuals with iSCI were able to follow the TW task, but were less capable of adapting gait to TW demands. CoM trajectory in TW correlated to functional scores (SCIM outdoor score), indicating that TW reflects challenging walking in daily environments. In addition, the finding of a correlation between CoM adaptability and NLI is novel, and points out that CoM is a potential comprehensive marker of spinal cord networks. Therefore, the investigation of CoM during TW in a larger age- and sex-matched cohort offers promising insight as to whether TW can be an effective method to characterize subtle gait disturbances and aid clinicians in tailoring personalized rehabilitation strategies.

In addition, longitudinal monitoring of TW may be used to investigate recovery mechanisms in individuals with iSCI, and potentially aid researchers in designing control systems for gait assist robotic devices for individuals with iSCI.

## Supplementary Information

**Additional file 1.** **Figure S1:** Relationship between task performance and CoM. (A) Accuracy in the ML direction improves with increase in vertical CoM trajectory length in participants with iSCI when performing TW (red dots) but is optimal in controls (gray triangles) without relation to CoM trajectory length. (B) Accuracy in AP direction is low in participants with iSCI (red dots) and controls (gray triangles) most likely due to inherent characteristics of the experimental TW task as the targets move in AP direction with preferred walking speed. AP accuracy improves with increase in AP-V CoM trajectory, this seems to be unrelated to iSCI pathology but rather due to experimental circumstances (inherent overshooting of target). Dots (participants) and triangles (controls) represent single participant and their ID. CoM = Center of mass; TW = Target walking; AP = anterior-posterior; ML = medio-lateral; V = vertical, iSCI = incomplete spinal cord injury. **Figure S2:** Cyclograms of knee-hip and knee-ankle demonstrating intra-limb coordination. Mean reference cyclograms of healthy controls are depicted for each subplot for NW (black solid line stance, dashed line swing) and TW (gray solid line stance, dashed line swing). (A) Knee-hip cyclograms for NW (participants with iSCI blue solid line stance, dashed line swing) and TW (participants with iSCI red solid line stance, dashed line swing) show a remarkable similarity between participants with iSCI and controls for majority of participants. Participants P01, P02, and P07, who clearly deviate from the normal pattern, have low self-selected walking speeds, indicating a cautious strategy to cope with the TW task. (B) Knee-ankle cyclograms show more variability in terms of limb coordination between controls and participants with iSCI. Most differences are found during swing phase (dashed lines) while the stance phase was similar for participants with iSCI and healthy controls (NW, darker shaded area; TW, light shaded area). Uniform differences between TW and NW typically occur at heel strike in the healthy while they are more variable in participants with iSCI. Note: joint angle data were not obtained in P15. Dots depict heel strike. Arrows indicate direction of movement. Cyclograms are clustered based on lesion level. TW = Target walking; NW = Normal walking; AP = anterior-posterior; ML = medio-lateral; V = vertical, iSCI = incomplete spinal cord injury; a.u. = arbitrary units.

## Data Availability

The datasets used and/or analyzed during the current study are available from the corresponding author upon reasonable request.

## Abbreviations

AP: Anterior–posterior
CoM: Center of mass
HS: Heel strike
iSCI: Incomplete spinal cord injury
IQR: Interquartile range
ISNCSCI: International standards for neurological classification of spinal cord injury
ML: Medio-lateral
MT2: Metatarsal 2
NLI: Neurological level of injury
NW: Normal walking
SCIM: Spinal cord independence measure
SD: Standard deviation
SPM: Statistical parametric mapping
TO: Toe off
TW: Target walking
V: Vertical
