# Supplementary material for: Mind your step: Target walking task reveals gait disturbance in individuals with incomplete spinal cord injury
Source: J Neuroeng Rehabil. 2022 Mar 25;19:36. doi: 10.1186/s12984-022-01013-7 (PMC8957135; doi:10.1186/s12984-022-01013-7)
Supplement: Supplementary file 1 — Additional file 1. Figure S1: Relationship between task performance and CoM. (A) Accuracy in the ML direction improves with increase in vertical CoM trajectory length in participants with iSCI when performing TW (red dots) but is optimal in controls (gray triangles) without relation to CoM trajectory length. (B) Accuracy in AP direction is low in participants with iSCI (red dots) and controls (gray triangles) most likely due to inherent characteristics of the experimental TW task as the targets move in AP direction with preferred walking speed. AP accuracy improves with increase in AP-V CoM trajectory, this seems to be unrelated to iSCI pathology but rather due to experimental circumstances (inherent overshooting of target). Dots (participants) and triangles (controls) represent single participant and their ID. CoM = Center of mass; TW = Target walking; AP = anterior-posterior; ML = medio-lateral; V = vertical, iSCI = incomplete spinal cord injury. Figure S2: Cyclograms of knee-hip and knee-ankle demonstrating intra-limb coordination. Mean reference cyclograms of healthy controls are depicted for each subplot for NW (black solid line stance, dashed line swing) and TW (gray solid line stance, dashed line swing). (A) Knee-hip cyclograms for NW (participants with iSCI blue solid line stance, dashed line swing) and TW (participants with iSCI red solid line stance, dashed line swing) show a remarkable similarity between participants with iSCI and controls for majority of participants. Participants P01, P02, and P07, who clearly deviate from the normal pattern, have low self-selected walking speeds, indicating a cautious strategy to cope with the TW task. (B) Knee-ankle cyclograms show more variability in terms of limb coordination between controls and participants with iSCI. Most differences are found during swing phase (dashed lines) while the stance phase was similar for participants with iSCI and healthy controls (NW, darker shaded area; TW, light shaded area). Unifor [file 12984_2022_1013_MOESM1_ESM.docx]

**Figure S1: Relationship between task performance and CoM.** (A) Accuracy in the ML direction improves with increase in vertical CoM trajectory length in participants with iSCI when performing TW (red dots) but is optimal in controls (gray triangles) without relation to CoM trajectory length. (B) Accuracy in AP direction is low in participants with iSCI (red dots) and controls (gray triangles) most likely due to inherent characteristics of the experimental TW task as the targets move in AP direction with preferred walking speed. AP accuracy improves with increase in AP-V CoM trajectory, this seems to be unrelated to iSCI pathology but rather due to experimental circumstances (inherent overshooting of target). Dots (participants) and triangles (controls) represent single participant and their ID. CoM = Center of mass; TW = Target walking; AP = anterior-posterior; ML = medio-lateral; V = vertical, iSCI = incomplete spinal cord injury.


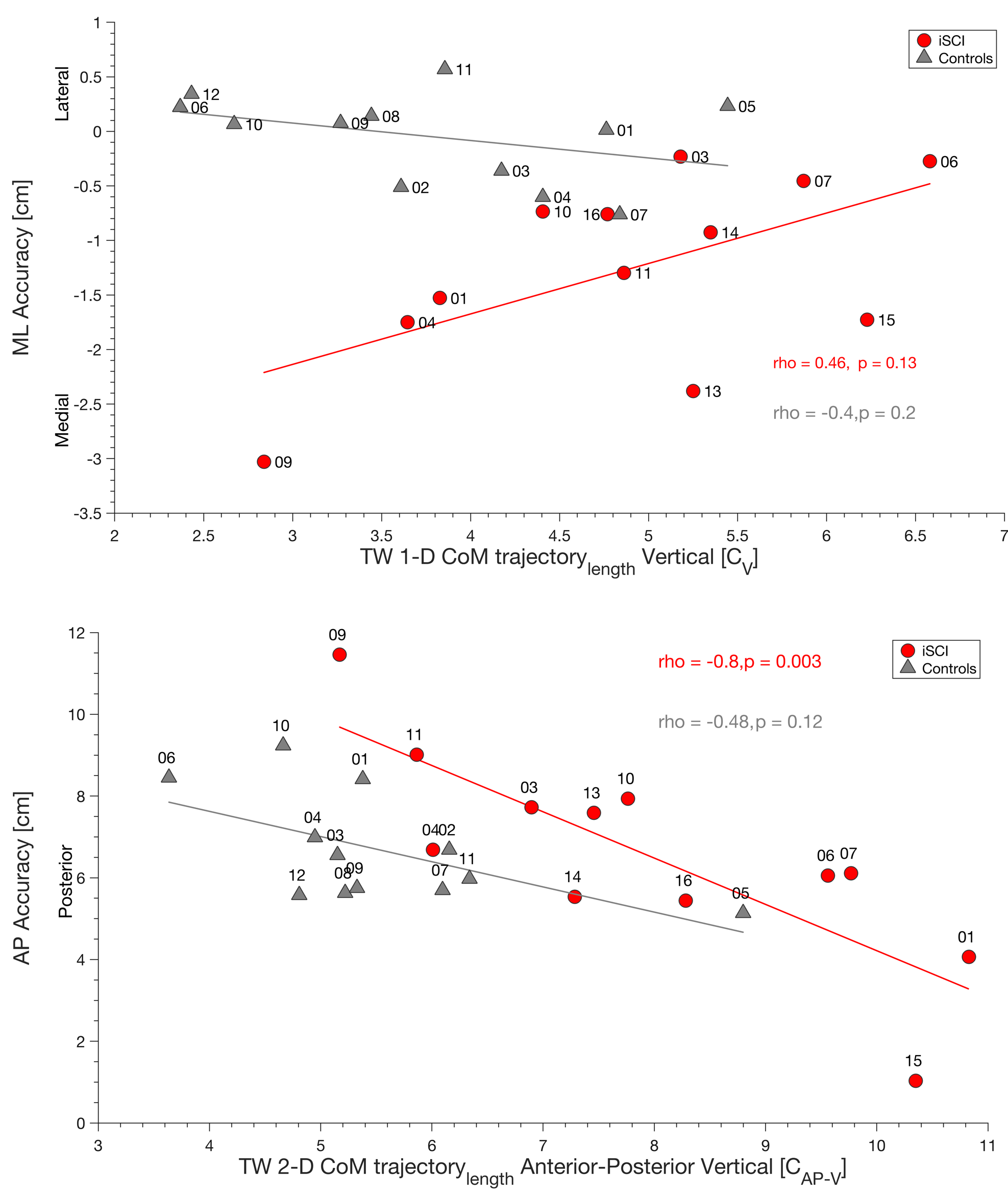

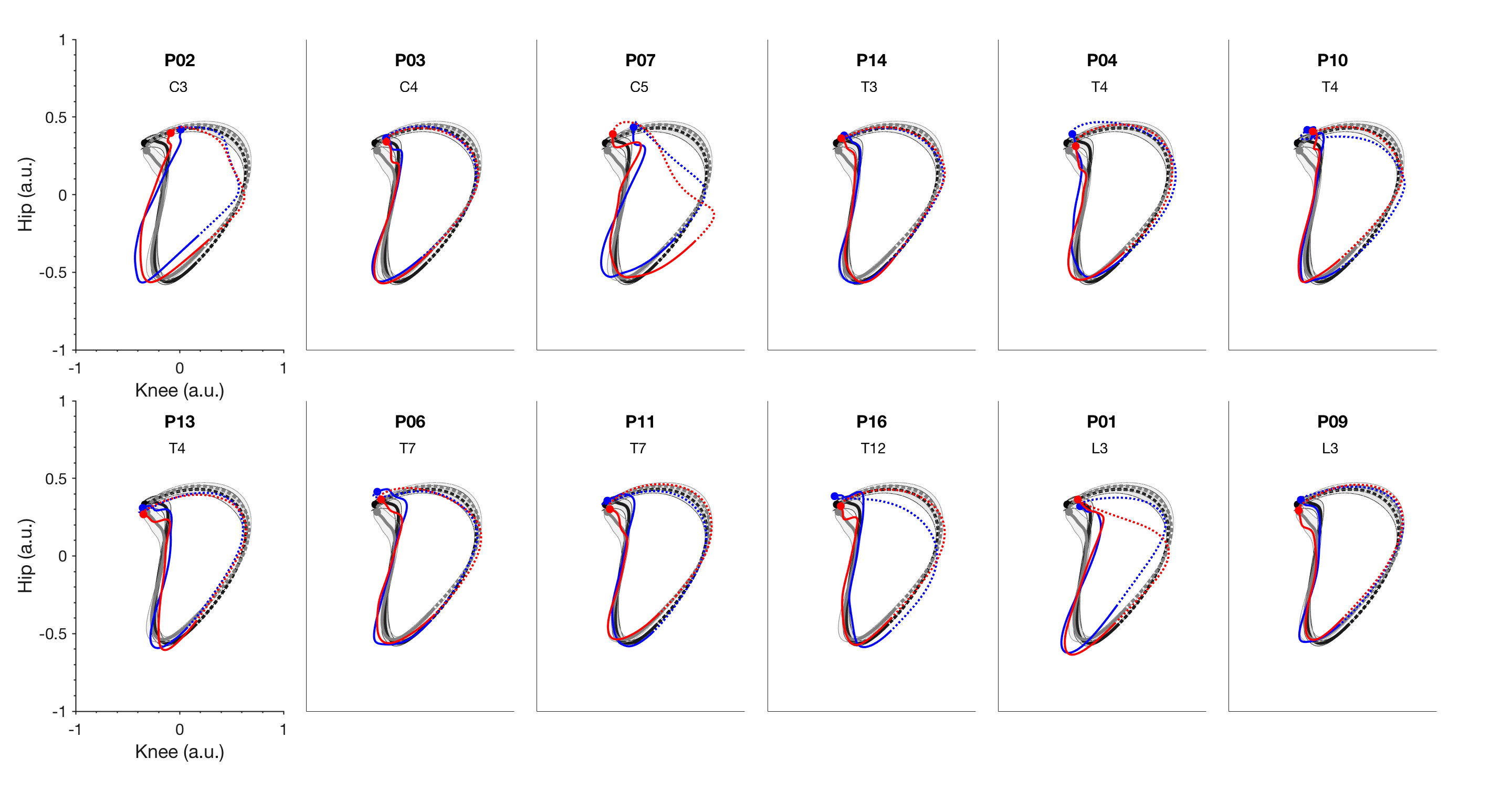

**Figure S2: Cyclograms of knee-hip and knee-ankle demonstrating intra-limb coordination.** Mean reference cyclograms of healthy controls are depicted for each subplot for NW (black solid line stance, dashed line swing) and TW (gray solid line stance, dashed line swing). (A) Knee-hip cyclograms for NW (participants with iSCI blue solid line stance, dashed line swing) and TW (participants with iSCI red solid line stance, dashed line swing) show a remarkable similarity between participants with iSCI and controls for majority of participants. Participants P01, P02, and P07, who clearly deviate from the normal pattern, have low self-selected walking speeds, indicating a cautious strategy to cope with the TW task. (B) Knee-ankle cyclograms show more variability in terms of limb coordination between controls and participants with iSCI. Most differences are found during swing phase (dashed lines) while the stance phase was similar for participants with iSCI and healthy controls (NW, darker shaded area; TW, light shaded area). Uniform differences between TW and NW typically occur at heel strike in the healthy while they are more variable in participants with iSCI. Note: joint angle data were not obtained in P15. Dots depict heel strike. Arrows indicate direction of movement. Cyclograms are clustered based on lesion level. TW = Target walking; NW = Normal walking; AP = anterior-posterior; ML = medio-lateral; V = vertical, iSCI = incomplete spinal cord injury; a.u. = arbitrary units.

NW iSCI

TW iSCI

NW Mean Controls

TW Mean Controls


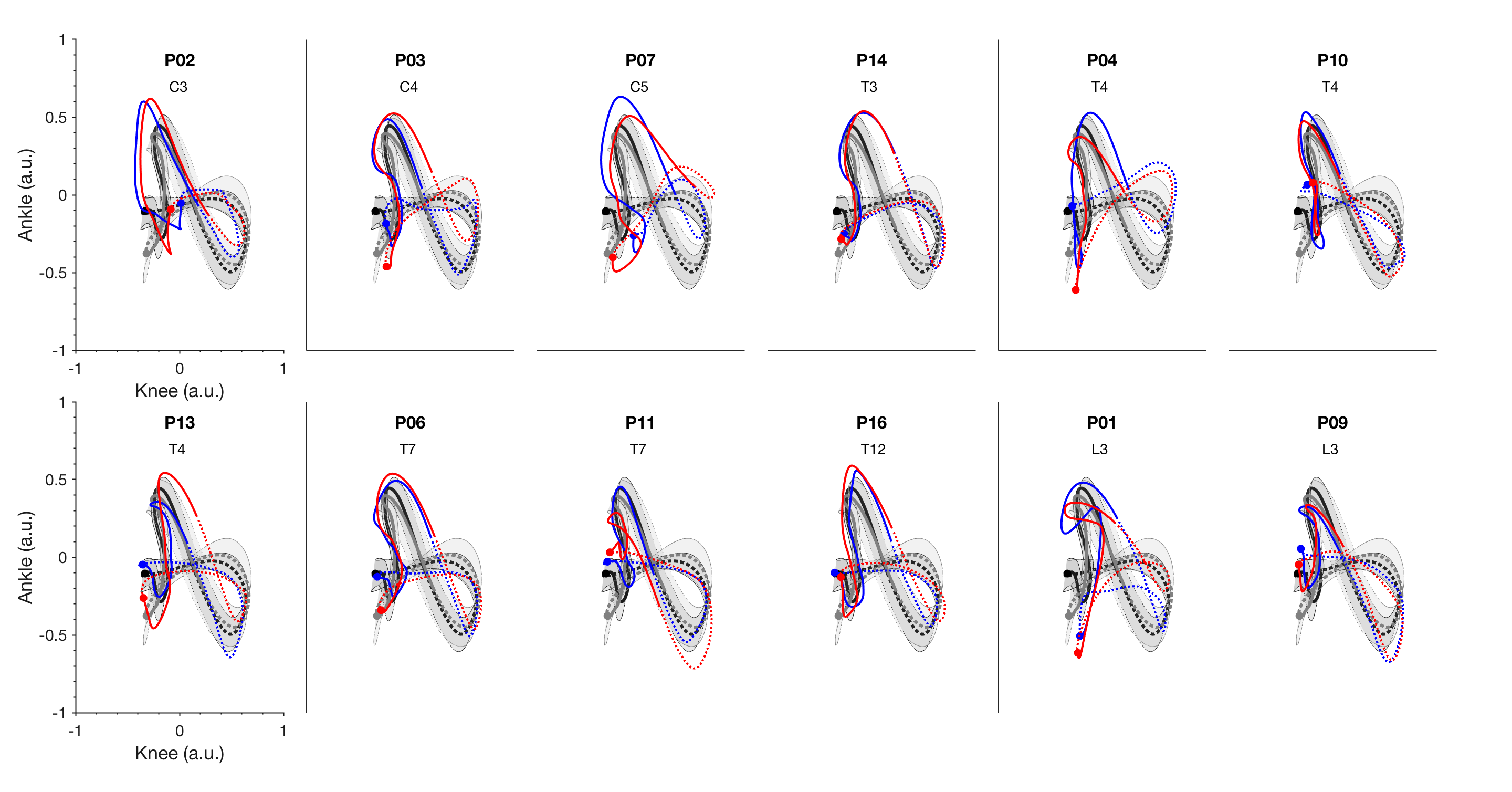


## *Results: Qualitative Analysis of intra-limb coordination during TW*

Joint angle cyclograms obtained for healthy controls showed distinct uniform differences between TW and NW tasks (suppl. Figure 2A-B, gray and black lines indicate means and variability of controls). Knee-hip cyclograms indicate distinct kinematic differences at heel strike when comparing NW and TW. They demonstrate that participants with iSCI are skilled walker’s despite of their higher age and independent of NLI, and severity of injury (suppl. Figure 2A, compare Table 1). Only participants P01, P02, and P07 deviated from the healthy controls. Interestingly, these are the participants who walked slower than the rest of the group (slower than 0.7 m/s, see Table 1). However, knee-ankle cyclograms (suppl. Figure 2B) demonstrated more variability also in other participants with iSCI compared to the healthy controls.

## *Discussion of the effects of intra-limb coordination: Qualitative interpretation of Cyclograms*

Intra-limb coordination has been demonstrated to be altered in participants with iSCI(1). During challenging walking tasks, such as obstructed walking and ground level changes, participants with iSCI compared to the healthy controls have been shown to increase knee flexion(2) and hip flexion(3), respectively. The knee-hip cyclograms of participants with iSCI resembled those of controls (suppl. Figure 2), except for those participants with iSCI with preferred walking speed below 0.7 m/s. In the TW condition, a systematic adaptation in controls during initial stance is obtained that is reduced in participants with iSCI. This corresponds to previous results in which hip-knee coordination was difficult to modulate for participants with iSCI(4, 5).

However, differences across participants with iSCI, compared to controls, were more obvious from the ankle joint cyclograms, suggesting that for TW the control of ankle joint movement was critical. This corresponds to previous reports, describing that ankle muscles provide essential proprioceptive information, and modulate CoM control, to ensure gait stability(6). In addition, skilled motor training has been shown to specifically improve hip-ankle coordination, further supporting the special role of ankle joint control in gait(7). Furthermore, ankle plantar-flexion was more variable because this movement was critical for hitting the target.

1. Awai L, Curt A. Intralimb coordination as a sensitive indicator of motor-control impairment after spinal cord injury. Front Hum Neurosci. 2014;8:148.

2. Ladouceur M, Barbeau H, McFadyen BJ. Kinematic adaptations of spinal cord-injured subjects during obstructed walking. Neurorehabil Neural Repair. 2003;17(1):25-31.

3. McFadyen BJ, Carnahan H. Anticipatory locomotor adjustments for accommodating versus avoiding level changes in humans. Exp Brain Res. 1997;114(3):500-6.

4. Easthope CS, Traini LR, Awai L, Franz M, Rauter G, Curt A, et al. Overground walking patterns after chronic incomplete spinal cord injury show distinct response patterns to unloading. J Neuroeng Rehabil. 2018;15(1):102.

5. Awai L, Bolliger M, Ferguson AR, Courtine G, Curt A. Influence of Spinal Cord Integrity on Gait Control in Human Spinal Cord Injury. Neurorehabil Neural Repair. 2016;30(6):562-72.

6. Sorensen KL, Hollands MA, Patla E. The effects of human ankle muscle vibration on posture and balance during adaptive locomotion. Exp Brain Res. 2002;143(1):24-34.

7. Malik RN, Eginyan G, Lynn AK, Lam T. Improvements in skilled walking associated with kinematic adaptations in people with spinal cord injury. J Neuroeng Rehabil. 2019;16(1):107.
